# 

**Published:** 1891-04-04

**Authors:** 


PRICE ONE PENNY.

No. 236, Vol. X.—April 4th, 1891.

[Registered at the General Post Office as a Newspaper for transmission in the United Kingdom and Abroad.]

WITH EXTRA NURSING SUPPLEMENT.

Per Annum, 6s. 6d.; post free.

Monthly Parts, 6d.; post free 8d.

Ditto per Annum, 8s., post free.

### THE HOSPITAL

GLASGOW WESTERN INFIRMARY — ST. THOMAS'S HOSPITAL — NORFOLK AND NORWICH HOSPITAL

A WEEKLY JOURNAL OF
Science, Medicine, Nursing and Philanthropy.

SATURDAY, APRIL 4th, 1891.

**A Vote of Confidence** . . . 1
**Passing Topics.**—Not all Condemned to Die . . . 2
**The Doctor's Armchair.**—Fifty Thousand Pounds . . . 3
**Poisons and Poisoners, I.**—Arsenic: Madeline Smith . . . 4
**Higher Education for Women in America** . . . 5
**Notes and News** . . . 6
**General Charities** . . . 7
**Scraps and Gleanings** . . . 7
**Annotations.**—Holiday Diet—Holiday Quiet—Holiday Exercise . . . 8
**Books Received** . . . 11
**The Practitioner's Mirror and Retrospect:—**
MEDICINE.—The Treatment of Peritonitis—Ulcerative Endocarditis . . . 9
SURGERY.—Trephining for Intracranial Pressure . . . 10
THERAPEUTICS.—Hydrastis Canadensis . . . 11
NEW DRUGS, APPLIANCES, AND THINGS MEDICAL—Antiseptic Dressings, &c. . . . 11
**The Student of Medicine**
**The Microscope in Medicine.** By FRANK J. WETHERED, M.D., XI.—The Examination of Urinary Deposits (*continued*) . . . 12

EXTRA SUPPLEMENT—THE NURSING MIRROR.

**En Passant.**—Gloucester District Nurses—Sheffield Infirmary—Rural Nursing Association—Cruelty—Bolton Nursing Association—Hospital for Women, Soho Square—A Disgrace to England—Short Items . . . i

**Lectures on Surgical Ward Work and Nursing.** By ALEXANDER MILES, M.B.Edin., C.M., F.R.C.S.E. Lecture XIX.—Special Operations . . . ii

**Princess Christian's Daughter** . . . ii

**Presentation** . . . ii

**The Eastern Hospital Scandal** . . . iii

**Examination Questions** . . . iii

**American News** . . . iv

**Everybody's Opinion.**—Pensions for Servants—Grumbling Probationers, iv; A Nurse's Hours . . . v

**Notes and Queries** . . . v

**Off Duty.**—The Celibates of the Future . . . vi

**Where to Go** . . . vi

**Amusements and Relaxation** . . . vi

